# Anaplastic Thyroid Carcinoma

**DOI:** 10.3389/fendo.2012.00084

**Published:** 2012-07-05

**Authors:** Augusto Taccaliti, Francesca Silvetti, Gioia Palmonella, Marco Boscaro

**Affiliations:** ^1^Division of Endocrinology, Azienda Ospedaliero Universitaria Torrette – AnconaAncona, Italy

**Keywords:** anaplastic, cancer, genetic alteration, prognosis, therapy, thyroid, treatment, tumor

## Abstract

Thyroid cancers represent about 1% of all human cancers. Differentiate thyroid carcinomas (DTCs), papillary and follicular cancers, are the most frequent forms, instead Anaplastic Thyroid Carcinoma (ATC) is estimated to comprise 1–2% of thyroid malignancies and it accounts for 14–39% of thyroid cancer deaths. The annual incidence of ATC is about one to two cases/million, with the overall incidence being higher in Europe (and area of endemic goiter) than in USA. ATC has a more complex genotype than DTCs, with chromosomal aberrations present in 85–100% of cases. A small number of gene mutations have been identified, and there appears to be a progression in mutations acquired during dedifferentiation. The mean survival time is around 6 months from diagnosis an outcome that is frequently not altered by treatment. ATC presents with a rapidly growing fixed and hard neck mass, often metastatic local lymph nodes appreciable on examination and/or vocal paralysis. Symptoms may reflect rapid growth of tumor with local invasion and/or compression. The majority of patients with ATC die from aggressive local regional disease, primarily from upper airway respiratory failure. For this reason, aggressive local therapy is indicated in all patients who can tolerate it. Although rarely possible, complete surgical resection gives the best chance of long-term control and improved survival. Therapy options include surgery, external beam radiation therapy, tracheostomy, chemotherapy, and investigational clinical trials. Multimodal or combination therapy should be useful. In fact, surgical debulking of local tumor, combined with external beam radiation therapy and chemotherapy as neoadjuvant (before surgery) or adjuvant (after surgery) therapy, may prevent death from local airway obstruction and as best may slight prolong survival. Investigational clinical trials in phase I or in phase II are actually in running and they include anti-angiogenetic drugs, multi-kinase inhibitor drugs.

## Epidemiology

The majority of thyroid malignancies are well-differentiated and have an excellent prognosis, on the other hand Anaplastic Thyroid Carcinoma (ATC) is an extremely aggressive cancer representing one of the most aggressive in humans with a dismal prognosis despite various therapeutic modalities. Although ATC accounts only for 2% of thyroid cancer incidence, it accounts for 14–39% of thyroid cancer deaths (Hundahl et al., 1998; Kitamura et al., 1999).

The female/male ratio is 5 to 1 and the peak of incidence is in the sixth and seventh decades of life (Kebebew et al., 2005). These observations are confirmed by other studies have demonstrated that about 68% of ATC patients were over 70 years of age and females constituted 70% and males 30% (Hundahl et al., 2000). The incidence of ATC is estimated at one to two cases per million population per year, and the trend has been decreasing even though the incidence of well-differentiated subtypes (e.g., papillary and follicular) of thyroid cancer has been increasing (Davies and Welch, 2006).

## Risk Factor

Risk factors for ATC are not well understood, in fact ATC might occur in patient without history of thyroid disease, or on the other hand, could have a history of goiter, or on histological examination, could co-exist differentiated thyroid cancer. In a study was demonstrated that 25% of the ATC patients had a prior history of thyroid goiter and another 10% with family history of goiter and it is also known that ATC is more common in places with endemic goiter as demonstrated by decreased incidence of ATC after iodine salt supplementation (Besic et al., 2010).

## Genetic Alterations

Recent advances in understanding the genetic and molecular pathogenesis of ATC hold promise for target therapy. ATC shows only few specific gene mutations, and the majority occurs in PTC (e.g., RAS and BRAF; Smallridge et al., 2009). This implies that many ATCs derive from preexisting PTCs by a process of dedifferentiation, acquiring new mutations like p53, catenin (cadherin-associated protein), beta 1, and PIK3CA (Nuocera et al., 2011). The remaining part of ATC may derive “*de novo*,” in this eventuality ATC is found alone in the removed thyroid (Nikiforov, 2004).

Genetic alterations in p53 gene are the most frequent in ATCs (55%), the other mutations follow for frequency as reported: RAS (22%), BRAF (26%), b-catenin (38%), PIK3CA (17%; Smallridge and Copland, 2010).

### p53 gene

It is a tumor suppressor gene located on chromosome 17p. P53 plays a key role in its nuclear transcription of factor production and in regulation of the cell cycle, DNA repair, and apoptosis. Mutations of p53 gene result in growth, angiogenesis, and dedifferentiation (O’Neill et al., 2010). Antico Arciuch et al. (2011) recently presented a paper with the characterization of the first mouse model of ATC, derived from the inactivation of p53 in cavies with constitutional activation of PI3CA.

### Braf

It is the most common mutation in papillary thyroid cancer and it is involved in tumor progression like radioactive iodine refractoriness and PTC recurrence (Nucera et al., 2010). BRAF mutations can be observed in both differentiated and undifferentiated tissues when ATCs co-exist with PTCs (Begum et al., 2004; Soares et al., 2004).

### Ras

It is a family of oncogenes important as cell growth regulators and they have a role in thyroid differentiation (Santarpia et al., 2008). The most important signal transduction pathway, for tumorigenesis, is the Ras-MAPK and phosphatidylinositol 3-kinase (PI3K)/Akt pathways (Luo et al., 2003).

### b-catenin and Wnt pathways genes

b-Catenin is a multifunctional protein and has two distinct functions. At first it binds as an intracellular stabilizer to cadherins to form the adherence junction. Dissociation of b-catenin from E-cadherin, mediated by various TKs, favors cell migration, and formation of metastases. At second, activation of Wnt signaling pathway stabilizes cytosolic b-catenin. This mechanism could be lost in presence on b-catenin mutations (Rao et al., 2006).

### PIK3CA

Among PI3Ks there are several classes, but the most important in human tumorigenesis and it is also the well characterized. It consist of heterodimers of a regulatory subunit and one of p110 catalytic subunits. The A and B types (respectively PIK3CA e PIK3CB) play an important role in human cancers (Xing, 2010). PIK3CA gene is located on 3q26.3 and its mutations are extremely rare in follicular or papillary thyroid carcinomas (Liu et al., 2008).

## Clinical Presentation

Clinically, ATC can present as a rapidly progressing disease invading surrounding local tissues and metastasizes to distant organs. Locally, ATC shows a rapidly enlarging anterior neck mass, with accompanying dysphagia (40%), voice change or hoarseness (40%), and stridor (24%). Regional symptoms included a noticeable lymph node mass (54%) and neck pain (26%). Systemic symptoms include anorexia, weight loss, and shortness of breath with pulmonary metastases. ATC is usually advanced at diagnosis and frequently surgically unresectable (Kebebew et al., 2005; Besic et al., 2010). Around 20–50% of patients present with distant metastases, most often pulmonary (Nuocera et al., 2011), and another 25% develop new metastasis during the rapid course of the disease [Lungs (80%), bone (6–16%), and brain (5–13%) were the most common sites of metastasis; Tennvall et al., 2002].

## Prognosis

The three different morphologic patterns identifiable at histologic analysis (squamoid, spindle cell, and giant cell) present similar biological and clinical features and none influences the prognosis (Hundahl et al., 1998).

Some factors as age, gender, tumor size, extent of disease, and resectability, influence clinical course and prognosis, however the median survival is usually less than 6 months, and death is either due to uncontrolled local invasion or distant metastasis. Younger female patients (<65 years old), with a small ATC (less than 5 cm or intra-thyroidal) and no distant metastasis at diagnosis, have a better prognosis (Rosai et al., 1993). Local disease consists in tracheal and esophageal invasion and obstruction (Pasieka, 2003; Kihara et al., 2004).

In an analysis of survival of ATC patients from the SEER database from 1983 to 2002, which included patients who survived for more than a month, the median survival was 4 months. On multivariate analysis, distant, or metastatic disease, tumor size greater than 7 cm, and treatment with surgery with or without radiotherapy were statistically significant prognostic markers with poor outcomes (*P* ≤ 0.05). Of interest, patients with extracapsular extension into adjacent tissue, the addition of radiotherapy was of benefit, on the contrary radiotherapy after surgery was of no benefit in patients who had disease confined to the thyroid or had distant metastasis (Sniezek, 2003). Age, sex, size of the tumor, resectability, and the extent of disease has been shown to affect the course of the disease (Chen et al., 2008). In a SEER-based study in the United States by multivariate analysis, only age less than 60 years, an intra-thyroidal tumor, and the combined use of surgical and external beam radiation therapy were identified as independent predictors of lower cause-specific mortality (McIver et al., 2001). In other series, female sex, tumor size less than 6 cm, age, and the extent of disease were the most favorable prognostic markers (Tan et al., 1995; Dziba et al., 2002). Among Koreans less than 60 years of age, tumor size less than 7 cm, and lesser disease burden were independent predictors of lower mortality (Lo et al., 1999). A recent study from France based on 26 patients with ATC, univariate analysis showed that age above 75, capsular invasion, lymph nodes metastasis, residual tumor after surgery, and lack of multimodal treatment (particularly radiotherapy in patients without tumor residue) are poor prognostic factors. Multivariate analysis in the same cohort showed age above 75, followed by node invasion, capsular invasion, and female sex to be poor prognosticators (Roche et al., 2010). In a study by Venkatesh and colleagues, patients with localized disease had a median survival of 8 months in comparison to 3 months for patients with metastatic disease (Tan et al., 1995; Lo et al., 1999; Kim et al., 2007; Roche et al., 2010). A prognostic index was developed by Sugitani et al. (2001) from a review of their series of 47 patients over 33 years (Venkatesh et al., 1990). Their index was based on a combination of four risk factors: (Hundahl et al., 1998; Kitamura et al., 1999) presence of acute symptoms, (Kebebew et al., 2005) tumor size greater than 5 cm, (Hundahl et al., 2000) distant metastasis, and (Davies and Welch, 2006) white cell count ≥10,000/μL (Venkatesh et al., 1990). Patients with a prognostic index less than or equal to one had a 62% survival rate at 6 months, whereas all patients with prognostic index of three and four died within 6 and 3 months, respectively.

## Treatment

Patients with ATC even in the absence of metastatic disease are considered to have systemic disease at the time of diagnosis. All ATCs are considered stage IV by the International Union Against Cancer (UICC) – TNM staging and American Joint Commission on Cancer (AJCC) system. Multimodality treatment consisting of surgery when feasible combined with radiation and chemotherapy is generally recommended.

### Surgery

The aim of surgery is to obtain a complete macroscopic resection, with microscopically clear resection margins. Complete resection has been identified as a prognostic factor in several clinical trials (Junor et al., 1992; Tan et al., 1995; Kobayashi et al., 1996; Sugitani et al., 2001). When feasible, surgery must aim at a radical intent. The categories of patients that may be most suitable for this approach are young patients (<65 years old) with small lesions (<6 cm) and no distant metastasis. However, surgery also plays an important role for palliation. Partial resection of the tumor followed by radiotherapy and chemotherapy may delay or avoid airway obstruction, although it can improve survival only by a few months (Nel et al., 1985). It is theoretically possible that, in selected patients, even in the setting of metastatic disease, surgery may result in an improved quality of life and prevent death from suffocation (Miccoli et al., 2007).

Since surgery alone is not able to control the disease even in patients with small intra-thyroidal masses, adjuvant therapy is always required, and can be administered either with radiotherapy (RT) or chemoradiotherapy. Whether surgery should be given up-front or after neoadjuvant treatment is a matter of debate. In fact, primary chemotherapy might make inoperable lesions operable, with the additional potential advantage of preventing distant metastasis. Moreover, Tennvall et al. (2002) reported encouraging results analyzing the outcome of 55 patients with ATC treated with neoadjuvant chemoradiotherapy between 1984 and 1999. The response to primary treatment was 72% (Yau et al., 2008).

### Systemic treatment

#### Cytotoxic agents

Anaplastic thyroid carcinoma cannot be regarded as a very chemo-sensitive tumor. Doxorubicin is not able to achieve more than a 20% response rate (Pacini et al., 1984). In a randomized study (Shimaoka et al., 1985) observed that combination chemotherapy based on doxorubicin (60 mg/m^2^) and cisplatin (40 mg/m^2^) was more effective than doxorubicin alone and provided a higher complete response rate. More recently, single drug docetaxel was tested as first-line chemotherapy in patients with advanced ATC. In a prospective phase II clinical trial of paclitaxel, 20 patients with metastatic ATC were enrolled and a remarkable response rate of 53% was obtained (Schoenberger et al., 2004). In a preclinical experiment (Schoenberger et al., 2004) only paclitaxel, gemcitabine, and vinorelbine appeared to be active in ATC (Bauer et al., 2003) and the combinations of vinorelbine/gemcitabine and paclitaxel/gemcitabine seemed to act synergistically. These results should receive confirmation in clinical trials.

#### Anti-angiogenetic agents

A common feature of thyroid cancers is their markedly increased vascularization, with an elevated expression of the vascular endothelial growth factor (VEGF) by immunohistochemistry, compared with normal thyroid tissue (Klein et al., 1999; Bauer et al., 2003). VEGF levels are correlated with stage, tumor size, nodal involvement, extra-thyroidal invasion, and distant metastases (Chaplin et al., 1996). On the basis of these findings, several drugs targeting angiogenesis have been evaluated against ATC.

- Combretastatin A4 phosphate (CA4P): is a tubulin-binding vascular disrupting agent that inhibits tumor blood flow. In contrast to other anti-angiogenetic drugs that block the formation of new vessels in tumors, vascular disrupting agents (such as CA4P) stop blood flow through already existing vessels, with the result of depriving tumor cells of oxygen and nutrients (McIver et al., 2001; Tozer et al., 2002). CA4P has activity against ATC cell lines and xenograft (Dowlati et al., 2002). In a phase I trial (Inai et al., 2004), one patient with ATC showed a progression-free survival of 30 months, however, the drug was found to be associated with significant cardiovascular side effects at the escalating doses employed.
- Axitinib (AG-013736) is an oral, potent, and selective inhibitor of VEGFRs 1, 2, and 3. Preclinical studies demonstrated that axitinib rapidly and selectively inhibits VEGF-dependent fenestrations and VEGFR-2 and 3 expression in endothelial cells, with the result of blocking angiogenesis and tumor blood flow (Baffert et al., 2006; Kamba et al., 2006; Mancuso et al., 2006; Mooney et al., 2009). The principal mechanism of action of axitinib is inhibition of VEGF signaling (Bauer et al., 2002).
- Bevacizumab (a monoclonal antibody anti VEGF) was tested alone and in combination with cetuximab in an *in vivo* model compared with doxorubicin. This study demonstrated that both drugs, either alone or in combination, inhibited tumor growth and angiogenesis better than doxorubicin (Prichard et al., 2007).
- AZD2171, a tyrosine-kinase inhibitor of the VEGFR-1 and VEGFR-2, blocked tumor growth and prolonged survival of ATC-bearing mice (Gomez-Rivera et al., 2007).

#### Histone deacetylase inhibitors

Histone deacetylase inhibitors are a promising class of antineoplastic agents that are able to induce cell differentiation, cell cycle arrest, and apoptosis through hyperacetylation of histones, with the potential to enhance the cytotoxicity of drugs such as doxorubicin. Preclinical studies have shown that valproic acid, a potent anti-convulsant agent, is able to enhance the activity of doxorubicin in cell lines derived from ATC alone or in combination with other drugs (Catalano et al., 2006; Kim et al., 2009).

#### Tyrosine-kinase inhibitors

- Imatinib (STI571) is an oral inhibitor of the ABL kinase (the product of the fusion of Bcr and Abl gene). In addition, it can specifically inhibit c-Kit and PDGF receptors, which are hyper-functioning in some malignancies. On the basis of the assumption that ATC which overexpresses PDGFR and/or Abl might respond to imatinib.
- Sorafenib (Bay43-9006, Nexavar) is an oral, small tyrosine-kinase inhibitor of the Raf protein kinase receptor, VEGFR-2, and PDGF-β and displays strong anti-angiogenetic activity. Sorafenib demonstrates an acceptable response rate in pre-treated ATC patients and further clinical studies are warranted.

#### Anti-EGFR agents

The epidermal growth factor receptor (EGFR) has been implicated in the pathogenesis of several types of cancer. There is supporting evidence that EGFR is expressed at high levels in ATC and papillary thyroid cancers (van der Laan et al., 1995). EGFR was expressed in all of the ATC cell lines examined and non-ligand dependent phosphorylation of EGFR was identified in half of the cell lines (Bergström et al., 2000). High expression of EGFR appears to be a negative prognostic factor in many types of tumors, but few studies have examined its prognostic role in thyroid cancers (Mizukami et al., 1991). Strong EGFR staining in papillary thyroid cancer was associated with poor prognosis (Akslen et al., 1993). These findings suggest that inhibition of EGFR may have anti-cancer efficacy in ATC.

- Gefitinib (ZD1839) is an orally active EGFR inhibitor that blocks EGFR-mediated downstream signal transduction. Preclinical trials have tested the activity of this drug against *in vitro* or *in vivo* models of ATC. Moreover Pennell et al studied the efficacy of gefitinib in a large group of thyroid cancer, including anaplastic thyroid cancer. Although gefitinib therapy did not result in any complete responses, the 32% of all patients underwent therapy with gefitinib have had reductions in tumor volume and prolonged stable disease, for the authors this may indicate biologic activity (Pennel et al., 2008).
- Cetuximab (C225) is a human-murine chimeric monoclonal antibody against EGFR. It has been approved by the Food and Drug Administration (FDA) for use in metastatic colorectal cancer and head and neck squamous cell carcinoma either metastatic or unresectable.

#### Agents targeting the NF-κB pathway

The 26s proteasome is a large ATP-dependent multimeric complex that degrades intracellular proteins that have been marked for proteolysis by the process of ubiquitination (Adams, 2004). The ubiquitin-proteasome pathway plays a significant role in neoplastic growth and metastatic spread. The proteasome is also required for activating nuclear factor κB (NF-κB) by degradation of its inhibitory protein factor κB inhibitor (I-κB). NF-κB is a transcription factor that upregulates a number of proteins involved in cancer progression including several anti-angiogenetic and anti-apoptotic factors (Aghajanian et al., 2005).

- Bortezomib (PS-341) is a proteasome inhibitor that has been approved by the FDA for the treatment of multiple myeloma and its mechanisms of action include the inhibition of I-κB, which leads to inactivation of the transcriptional factor NF-κB (Davis et al., 2004; Papandreou et al., 2004). NF-κB is often constitutively activated in medullary thyroid carcinoma and ATC, and is therefore implicated in their pathophysiology (Pacifico et al., 2004). Bortezomib has also been shown to increase the expression of TRAIL (TNF-related-apoptosis-induced-ligand) receptors (TRAIL-R1 and 2) and to sensitize tumors to TRAIL-mediated killing (Conticello et al., 2007). The high cytotoxic activity and good *in vivo* tolerability of bortezomib holds promise for its future use in the treatment of ATC patients.

#### Agents targeting farnesyl-transferase

A new group of therapeutic agents called farnesyl-transferase inhibitors (FTIs) has been used in the treatment of solid tumors. Activating Ras mutations are common in thyroid cancers (Wynford-Thomas, 1997). Ras, the protein product of the Ras proto-oncogene, requires post-translational modification by conjugation of a farnesyl moiety to its C-terminal amino-acid. After farnesylation, Ras is localized to the inner surface of the cell membrane and is able to transduce the mitogenic signals mediated by tyrosine-kinase receptors. Farnesylation-blocking agents therefore operate by inhibiting Ras activity.

- Manumycin A is a natural product of *Streptomyces parvulus* that inhibits farnesyl-transferase and has antitumor activity against a variety of cancers *in vitro* and in xenograft models (Ito et al., 1996; Nagase et al., 1996). Apart from inhibition of angiogenesis, manumycin A causes apoptosis by inducing the pro-apoptotic protein Bax (Pan et al., 2005). No clinical trials have been performed to determine the activity and/or efficacy of manumycin A against ATC.

#### Proteasome inhibitor

PI3 kinase and MAPK pathways bind to heat shock protein 90 (HSP90). Disruption of HSP90 lead to reduced cell signaling and to cell death. Many cancer’s cells demonstrated an overactivation of HSP90, so HSP90 inhibitors have been developed (Braga-Basaria et al., 2004).

- Geldanamycin is a benzoquinoid ansamycin antibiotic that works via the dissociation of the HSP90, affecting the stability and the steady state level of these oncoproteins. Park et al. (2003) demonstrated *in vitro* that geldanamycin inhibits thyroid cancer cell proliferation, down-regulates oncoproteins, and inhibits EGF-induced invasion. However Geldanamycin is weighed by important hepatic toxicity (Neckers, 2002).
- 17-Allylamino-17-demethoxygeldanamycin (17-AAG) 17-AAG maintains similar antitumor properties of geldanamycin but has fewer associated side effects. 17-AAG binds the ATP-binding pocket in the amino terminus of Hsp90, thereby inhibiting Hsp90 function (Neckers, 2002). Braga-Basaria and colleagues evaluated *in vitro* the activity of 17-AAG on cancer cells. Of the various cancer’s cells lines, the most enhanced pro-apoptotic effects was demonstrated for those cell lines with highest level of HSP 90; thus, although not clearly applied yet to anaplastic thyroid cancer cells, the data suggest that HSP90 levels may serve as a biomarker for 17-AAG activity (Braga-Basaria et al., 2004).

#### Agents targeting matrix metalloproteinases

Matrix metalloproteinases (MMPs) are an important group of enzymes mediating the endothelial cell invasion and migration required for the formation of new capillaries, a crucial step in the angiogenesis process.

- Minocycline is a semi-synthetic analog of tetracycline active against MMPs through chelation of the zinc ion at the active site of the enzyme. In a preclinical study, She and Jim (2006) investigated the effect of adding minocycline to manumycin A and paclitaxel against human ATC cells xenografted in nude mice, and demonstrated that the triple-drug combination resulted in the lowest average tumor growth rate, yielding significantly better survival than manumycin A alone, paclitaxel alone, or manumycin A plus paclitaxel. This novel combination deserves further investigation for the treatment of ATC.

#### Agents targeting PPARγ

Peroxisome proliferator-activated receptor gamma (PPARγ) agonists have demonstrated antitumor activity against a variety of human cancers in preclinical models and clinical trial (Zang et al., 2003). The mechanism of action of the different classes of these compounds, which comprise non-steroidal anti-inflammatory drugs, amino-acid derivatives, polyunsaturated fatty acids, eicosanoids, and thiazolidinediones, is attributed to the capacity of binding and activating PPARγ. PPARγ acts as a tumor suppressor gene, upregulating important enzymes which control the cell cycle (Nakajima et al., 2001).

Thiazolidinediones represent the most widely investigated pharmaceutical class among PPARγ agonists (Aiello et al., 2006). In a preclinical study, two agents belonging to this class, ciglitazone and rosiglitazone, showed promising biological effects in ATC cells, such as an increased rate of apoptosis and inhibition of anchorage-dependent and -independent growth and migration. Furthermore, rosiglitazone increased the expression of thyroid-specific differentiation markers, thus inducing a partial reversion of the epithelial-mesenchymal transition in ATC cells, which correlates with ATC growth and dissemination (Weng et al., 2006).

RS5444 is another thiazolidinedione agent and a PPARγ agonist. RS5444 demonstrated antitumor activity in preclinical studies, with a mechanism which includes the transactivation of genes regulating cell proliferation, apoptosis, and differentiation. In particular, PPARγ activation is able to upregulate p21 protein, which is known to complex and inhibit an heterodimeric complex called cyclin dependent kinase 2 (CDK2)-cyclin E/A, responsible for cell cycle progression. Cells expressing nuclear p21 are subsequently arrested in the G0–G1 phase of the cell cycle (Pei and Xiong, 2005).

### Radiation

Radiation does not alter the course of ATC in most patients. On the other hand, when combined with surgery and chemotherapy, it can prolong the short-term survival in select and subset of patients. Intensity-modulated radiation therapy (IMRT) based on computerized treatment planning and delivery is able to generate a dose distribution that delivers radiation accurately with sparing of the surrounding normal tissue (Rosenbluth et al., 2005; Lee et al., 2007). Higher doses of radiation can be given over a shorter time with less toxicity by employing hyperfractionation techniques (Tennvall et al., 1990; Wong et al., 1991). Toxicity can be a limiting factor with radiation. Kim and Leeper (1987) reported complications particularly, pharyngitis, esophagitis, and tracheitis in their series. Wong et al. (1991) also noted skin changes, esophageal toxicity, and radiation myelopathy. Daily doses of greater than 3Gy should be cautiously used as it can increase the incidence of myelopathy (Wong et al., 1991).

More encouraging are the results reported by the concurrent use of taxanes and radiation. After standard dose of 60Gy in 30 fractions along with docetaxel 100 mg every 3 weeks for six cycles, an improvement of disease with partial remission (33%) and complete response (64%) was observed in ATC patients (Troch et al., 2010; Brierley, 2011).

## Conflict of Interest Statement

The authors declare that the research was conducted in the absence of any commercial or financial relationships that could be construed as a potential conflict of interest.
